# Characterization of the Spatial and Temporal Expression of Two Soybean miRNAs Identifies SCL6 as a Novel Regulator of Soybean Nodulation

**DOI:** 10.3389/fpls.2019.00475

**Published:** 2019-04-16

**Authors:** Md Shakhawat Hossain, Nhung T. Hoang, Zhe Yan, Katalin Tóth, Blake C. Meyers, Gary Stacey

**Affiliations:** ^1^C.S. Bond Life Science Center, Divisions of Plant Sciences and Biochemistry, University of Missouri, Columbia, MO, United States; ^2^Donald Danforth Plant Science Center, St. Louis, MO, United States

**Keywords:** miRNA, miR171, GRAS TF, *Scarecrow like-6*, NSP2, nodulation, symbiosis, soybean

## Abstract

MicroRNAs (miRNAs) control expression of endogenous target genes through transcript cleavage or translational inhibition. Legume plants can form a specialized organ, the nodule, through interaction with nitrogen fixing soil bacteria. To understand the regulatory roles of miRNAs in the nodulation process, we functionally validated gma-miR171o and gma-miR171q and their target genes in soybean. These two miRNA sequences are identical in sequence but their miRNA genes are divergent and show unique, tissue-specific expression patterns. The expression levels of the two miRNAs are negatively correlated with that of their target genes. Ectopic expression of these miRNAs in transgenic hairy roots resulted in a significant reduction in nodule formation. Both gma-miR171o and gma-miR171q target members of the GRAS transcription factor superfamily, namely *GmSCL-6* and *GmNSP2*. Transient interaction of miRNAs and their target genes in tobacco cells further confirmed their cleavage activity. The results suggest that gma-miR171o and gma-miR171q regulate *GmSCL-6* and *GmNSP2*, which in turn, influence expression of the *Nodule inception* (*NIN*), *Early Nodulin 40* (*ENOD40*), and *Ethylene Response Factor Required for Nodulation* (*ERN*) genes during the *Bradyrhizobium japonicum*-soybean nodulation process. Collectively, our data suggest a role for two miRNAs, gma-miR171o and gma-miR171q, in regulating the spatial and temporal aspects of soybean nodulation.

## Introduction

The agricultural and ecological success of legume species is largely due to their ability to form a mutualistic relationship with rhizobium bacteria. This beneficial microbe–plant symbiosis results in the formation of a specialized organ, the root nodule, which rhizobium bacteria colonize resulting in the conversion of atmospheric N_2_ into NH_3_, a form of nitrogen that can be readily utilized by the plants. This biological nitrogen fixation (BNF) allows legume plants to thrive in N-deficient soils without the necessity of N fertilizer addition. The initiation of the intimate symbiosis between rhizobium and host plant involves an exchange of diffusible chemical signals. The host plants release flavonoids in root exudates to attract rhizobia (Senthil et al., 2006; Sugiyama et al., 2008) and induce gene expression in the rhizobia. For example, the isoflavones genistein and daidzein induce the expression of the nodulation genes of *Bradyrhizobium japonicum* (*B. japonicum*) (Banfalvi et al., 1988). Induction of the rhizobial *nod* genes leads to the synthesis of the lipochitooligosaccharide nodulation factor (i.e., *nod* factor) that is recognized by the plant inducing key events in the infection process (Stacey et al., 2006; Oldroyd, 2013; Liang et al., 2014). Establishment of a successful legume-rhizobium symbiosis requires the successive activation of both symbiont and host genes in a spatially and temporarily correlated manner (Oldroyd, 2013). It is now known that regulation of symbiotic development requires the action of a variety of regulatory factors, including a number of microRNAs (miRNAs).

microRNAs are 21 to 24 nucleotides in length, are often highly conserved non-coding RNA molecules, and they regulate the expression of their target genes either by translational repression or mRNA cleavage (Llave et al., 2002; Brodersen et al., 2008). In both plants and animals, miRNAs are involved in a variety of biological and metabolic processes including but not limited to defense against viruses and regulation of gene expression during development (Carrington and Ambros, 2003), organ development, and stem cell differentiation (Zhang et al., 2007). Especially in plants, miRNAs are crucial in controlling tissue differentiation and development, signal transduction, vegetative to reproductive growth transition, and response to biotic and abiotic stress (Zhang et al., 2008). Unlike human miRNAs, most plant miRNA genes are located inside intergenic regions between two adjacent genes and transcriptionally regulated by their own promoters and terminators (Tang, 2010).

A number of miRNAs are known to control various stages of the soybean-rhizobium symbiosis. In our recent publication, we studied fifteen soybean small RNA libraries derived from nodules at different developmental stages including 10, 15, 20, 25, and 30-days post-infection (DPI). We identified 139 miRNAs that were differentially regulated during nodule development (Yan et al., 2015). A similar approach was used by the Xia Li group (Wang et al., 2009) in which they prepared small RNA libraries from mature nodules harvested 28 days after *B. japonicum* inoculation and identified 20 soybean-specific miRNAs. In an earlier publication, Subramanian et al. (2008) identified miRNAs involved in soybean nodulation identified from libraries derived from roots 3 h after *B. japonicum* inoculation, which resulted in the identification of 20 conserved as well as 35 novel miRNAs (Subramanian et al., 2008). These surveys of soybean miRNAs expressed during nodulation identified several miRNAs whose expression changes in response to *B. japonicum* inoculation. For example, the abundances of miR159 and miR393 increase in response to inoculation, while those of miR160 and miR169 decrease. While some miRNAs appear to respond early during the infection process, the expression of others suggests a role in controlling functions in mature nodules. Examples include miR167, miR171, miR396, miR399, and miR1507 to miR1510 (Simon et al., 2009). The mRNA targets of these miRNAs can be transcription factors, such as in the case of miR172 (Yan et al., 2013; Wang et al., 2014). Yan et al. (2013) reported that miR172 regulated expression of an *APETALA2* (*AP2*) transcription factor that appeared to control the expression of non-symbiotic hemoglobin, which showed a positive correlation with nodule number. Wang et al. (2014) found that miR172c targets a repressive *AP2* TF, which directly binds to the promoter of early nodulin gene *ENOD40* and activates gene expression to regulate nodule initiation. miRNA can also target *Auxin Response Factors* (*ARF*). For example, miR167 targets *GmARF8a* with a role in regulating nodule and lateral root number (Wang et al., 2015).

The miR171 family, 21 nucleotides in length, is highly conserved among angiosperm plants (Zhu et al., 2015). The number of miR171 genes varies among various plant species. *Arabidopsis*, for example, has three copies (i.e., miR171a, b, and c). Barley, *Brachypodium*, rice, and maize have two, four, nine, and 14 miR171 isoforms, respectively (Curaba et al., 2013). There are 21 soybean miR171 genes listed in miRBase identified by sequencing small RNA libraries derived from a variety of tissues, including nodules (Yan et al., 2015). miR171 is best known to regulate various plant growth and development processes by targeting members of the GRAS-domain transcription factor family. The name of this family is derived from the *GAI* (*GIBBERELLIC ACID INSENSITIVE*), RGA (*REPRESSOR of GAI*), and SCR (*SCARECROW*) transcription factors (Fiorilli et al., 2015). The two best studied targets of miR171 are *SCARECROW*-LIKE (*SCL*) and *NODULATION SIGNALING PATHWAY 2* (*NSP2*) transcription factors (Hirsch et al., 2009; Hofferek et al., 2014; Zhu et al., 2015). In *Arabidopsis*, miR171 targets three *SCL6* genes, including *SCL6-II*, *SCL6-III*, and *SCL6-IV*, also known as *HAIRY MERISTEM* (*HAM*) and *LOST MERISTEMS* (*LOM*), which are critical for shoot apical meristem and axillary meristem maintenance. Overexpression of miR171 precursors altered shoot architecture in *Arabidopsis*, by inhibiting axillary bud formation and suppressing flower number (Schulze et al., 2010). In addition, miR171 also regulates chlorophyll biosynthesis by targeting *SCL6*, *SCL22*, and *SCL27* mRNAs. *SCL27* controls expression of *PROTOCHLOROPHYLLIDE OXIDOREDUCTASE C* (*PORC*), which is a light-inducible enzyme important for photosynthesis (Pattanayak and Tripathy, 2011). In monocots, such as barley (*Hordeum vulgare* L. cv. Golden promise), ectopic overexpression of Hvu pri-miR171a resulted in pleiotropic effects such as dwarf stems with short internode length, delayed flowering time, and partially sterile spikes (Curaba et al., 2013).

Earlier reports demonstrated that miR171 targets transcripts of the NSP2 transcription factor in *Lotus japonicus* and *Medicago truncatula*, which is an important regulator of nodulation and mycorrhization (Oldroyd, 2013; Hofferek et al., 2014). In the current work, we describe additional layers of regulatory complexity by demonstrating that, in soybean, the temporal and spatial expression of two distinct miR171 genes (namely, gma-miR171o and gma-miR171q) controls soybean nodulation by regulating expression of both GmNSP2 and the additional GRAS family transcription factor *GmSCL-6*, which previously had not been known to play a role in nodulation.

## Materials and Methods

### Plant Materials, Conditions and Bacterial Strains

In this study, we used soybean and tobacco as research materials. For soybean (*Glycine max*), seeds of cultivar Williams 82 were surface sterilized, germinated and grown in a vermiculite: perlite soil mixture (3:1 ratio) in a growth chamber with a 16-h light and 8-h dark photoperiod, set to 23°C during the dark and 26°C during the light regime at 80% humidity. At the time of sowing seeds, the plants were inoculated with *B. japonicum* strain USDA110. Based on the experimental needs, we used either wild type, GUS or LacZ tagged *B. japonicum* USDA110. *B. japonicum* cells were cultured in HM medium (Cole and Elkan, 1973) with the appropriate antibiotics, and grown at 30°C for 3 days. After 3 days of culture, bacteria were pelleted, washed two times with sterilized deionized (DI) water, and diluted in sterilized DI water to OD_600_ of 0.8 for inoculation. Plants were supplied with B&D (Broughton and Dilworth) (Broughton and Dilworth, 1971) with no nitrogen nutrient solution or with supplement of 0.5 mM NH_4_NO_3_ as in the case of non-infected experiments. Tissues such as roots, nodules or transgenic hairy roots were collected from different experiments in this study for statistical analysis and/or frozen immediately in liquid nitrogen and stored at -80°C for further use. Each experiment was done with three biological replicates.

Tobacco (*Nicotiana benthamiana*) leaves were used for a transient expression system to confirm miRNA-target interaction. We grew tobacco plants in Sunshine soil (Sungro Horticulture) in a growth chamber until the first two to three leaves were fully expanded. At this stage, tobacco leaves were used for co-infiltration.

### RNA Extraction, cDNA Synthesis, and qRT-PCR Analysis

For mature miRNAs, miRNA precursors, and gene expression analysis, total RNA was isolated using Trizol Reagent according to the manufacturer’s instructions (Invitrogen, Carlsbad, CA, United States), and subsequently purified using chloroform extraction from various soybean root tissues as described in results. cDNA synthesis was carried out as described previously (Yan et al., 2013). Primers used for this study are shown in Supplementary Table S1. qRT-PCR was performed as described (Libault et al., 2008), and relative expression data were analyzed following the methods of our previous publication (Libault et al., 2010). Student’s *t*-test was used to compare differences between control and experimental values. For any expression analysis, three biological replications were used. Stem loop mature miRNA and precursor qRT-PCR analyses were carried out as described previously (Yan et al., 2015). In order to measure expression of miRNA primary transcript, specific primers within the miRNA primary sequence were designed for regular qRT-PCR. For the detection and quantification of mature miRNA abundance, we conducted stem-loop RT PCR with a stem-loop-specific RT primer containing six nucleotide specific sequences from the complementary 3′ end sequence of each miRNA. 1 μg of gDNA-free RNA was used as the template for cDNA synthesis by Superscript III (Invitrogen). Subsequently, a pair of miRNA-specific forward primer and universal reverse primer was used to amplify the mature miRNA-containing sequence from the cDNA template using SYBR Green PCR mix (ABI enzyme) (Varkonyi-Gasic et al., 2007). Two reference genes, snoR1 and 5.8S were used to determine the relative expression of each miRNA.

### Promoter Cloning and Expression Analysis

The cloning of the promoter regions for the various miRNA genes involved PCR amplification of 2154 bp 5′ of the precursor of gma-miR171o (miRBase accession, MI0019749) and a 2119 bp fragment 5′ of the gma-miR171q (miRBase accession, MI0019760) precursor sequence. Likewise, additional promoter regions were cloned using a 2533 bp fragment 5′ of *pGmNSP2.1* (-73 bp from ATG) and 2485 bp 5′ of *pGmSCL6-1* (-2 bp from ATG). The PCR primers used are listed in Supplementary Table S1. *PspxI* and *AscI* restrictions sites were added to the 5′ end of these primers to allow cloning into the modified vector, named “*pSoyGUS*.” The vector *pSoyGUS* was modified from *pCAMGFP-CvMV:GWox* (Invitrogen). The vector *pCAMGFP-CvMV:GWox* has a *GFP* selection marker for transgenic events and contains a strong CvMV (Cassava Mosaic Virus) promoter. For the construction of *pSoyGUS*, the *GUS* gene was introduced by Gateway cloning (Invitrogen). To facilitate *GUS* activity driven by a native promoter, the CvMV promoter was removed from *pSoyGUS* using *PacI* digestion and then re-ligated. The various native promoters (i.e., gma-miR171 o and gma-miR171q, *GmSCL6-1* and *GmNSP2-1*) were then introduced using *PspxI* and *AscI* restrictions sites in the *pSoyGUS* vector just before and after the *PacI* sites. The fidelity of the various cloned products was confirmed by DNA sequencing. An empty pSoyGUS vector which contained the GUS gene driven by CvMV promoter was used as a positive control for GUS gene expression.

### Plasmid Vector Construction

Ectopic expression of the various miRNA involved PCR amplification from soybean genomic DNA of the miRNA precursor fragments containing ∼200 bp upstream and downstream of the mature miRNAs, gma-miR171o and gma-miR171q. In addition, full-length cDNA fragments of *GmNSP2.1* (Glyma04g43090) and *GmSCL6-1* (Glyma01g18040) were amplified from soybean root/nodule cDNA using primers as shown in Supplementary Table S1. The resulting miRNA precursors and target gene fragments were cloned into the pDONR/Zeo vector (Thermo Fisher) and subsequently, recombined using Gateway^®^ BP and LR Clonase^®^ II enzyme mixes (Invitrogen) into *pCAMGFP-CvMV:GWox* binary vector, which has a strong CvMV promoter and a GFP reporter cassette for transgene selection.

Mutations in the various miRNAs and their target genes were introduced by PCR amplification. Two sets of primers covering the mature miRNA sequence were used to modify key residues of the miRNA sequences specifically at the potential target cleavage sites. Similarly, the target genes were mutated specifically at those sites cleaved by the miRNAs. Amplified fragments were cloned into the *pDONR/Zeo* vector and later into the *pCAMGFP-CvMV:GWox* binary vector.

For the target gene RNAi constructs, ∼103 bp of *GmNSP2.1* and ∼135 bp of *GmSCL6-1* gene specific fragments were amplified using primers described in Supplementary Table S1. Amplified fragments were cloned into the *pDONR/Zeo* vector and the resulting positive plasmids were recombined into the *pCAMGFP-CvMV-GWi* binary vector (Graham et al., 2007) using the Gateway^®^ LR Clonase^®^ II enzyme mixes (Invitrogen).

### Transient Interaction of miRNAs and Their Target Genes

For transient interaction, we used *N. benthamiana* at the stage when the first two to three leaves were fully expanded. All the positive binary vectors were transferred into *Agrobacterium* GV3101 and co-infiltrated according to the procedure described previously (Li, 2011). miRNA (gma-miR171o and gma-miR171q) overexpression constructs (with or without mutation) and their corresponding target genes (*GmSCL6-1* and *GmNSP2.1*) that fused to GFP were co-infiltrated at the abaxial leaf surface following protocols as described previously (Li, 2011) and transient interaction assayed 24, 48, and 72 h after co-infiltration and the results were documented.

### Soybean Hairy Root Transformation and Microscopy

For miRNA and target gene expression, target gene RNAi, as well as expression localization studies in soybean, the appropriate plasmid constructs as mentioned above were transformed into *Agrobacterium rhizogenes* K599 and used for hairy root transformation as previously described (Kereszt et al., 2007). Transgenic hairy roots were selected using a dissecting fluorescence microscopy based on constitutive GFP expression. The phenotypes measured for each construct are detailed in the results section. Transgenic tissues (root and nodule) were harvested, frozen in liquid nitrogen, and used for subsequent downstream analyses. To understand and analyze the nodule morphology and structure, the nodules were fixed in 4% paraformaldehyde, 3% glutaraldehyde in sodium phosphate buffer and embedded in paraplast. Nodule section was stained with toluidine blue (0.1%) following by washes with water, and then dried at room temperature before microscopic examination.

For statistical analysis, multiple experiments were carried out, and in each experiment setup, we used at least 20 plants per replicate for quantification of nodule formation. Student’s *t*-test was used for statistical analysis.

## Results

### Characteristic Features of miR171 in Soybean

The miR171 family is highly conserved among various plant species (Zhu et al., 2015), and plays a diverse role in plant growth and development, including the legume N_2_-fixing symbiosis (Subramanian et al., 2008; Bazin et al., 2012; De Luis et al., 2012; Hofferek et al., 2014). In our earlier publication, we reported that miR171 targets the *Nodulation Signaling Pathway 2* (*NSP2*) gene in soybean based on Parallel Analysis of RNA Ends (PARE) data (Arikit et al., 2014; Yan et al., 2015). However, a detailed functional role for miR171 and its target genes in soybean was not reported. Therefore, to begin such a study, we first searched miRBase database^1^ and the soybean genome to identify all predicted miR171 family members. In total, 21 miR171 family members (i.e., gma-miR171 paralogs) were identified in soybean (Supplementary Figure S1). Phylogenetic analysis of these 21 primary transcripts (Supplementary Figure S1A) indicates that they are the products of unique genes. Although encoded by distinct miRNA genes, some members (such as gma-miR171o and gma-miR171q) have identical 21 nt mature miRNA sequences (Supplementary Figure S1B). Hence, it became of interest to us to explore the function of these identical miRNAs derived from distinct primary transcript sequences, especially since subsequent analysis showed that both are involved in the regulation of soybean nodulation.

### Gma-miR171o and Gma-miR171q Exhibit Distinct Expression Patterns in Response to Bacterial Infection

**FIGURE 1:**
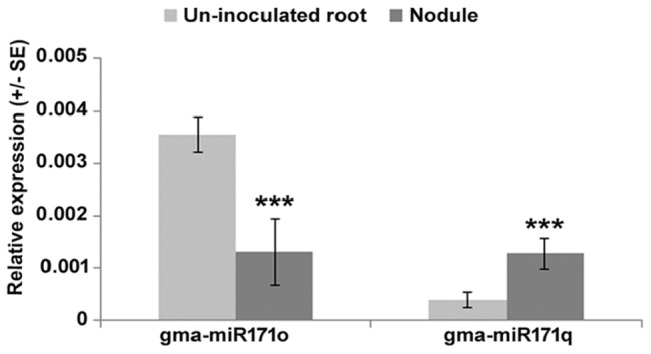
Expression patterns of gma-miR171o and gma-miR171q during symbiosis with *B. japonicum*.

To understand the symbiotic role of gma-miR171o and gma-miR171q in soybean, we measured the relative expression level of these two miRNAs in nodules 3 weeks post-inoculation with *B. japonicum*, as compared to uninfected root tissues (Figure 1). Interestingly, these two miRNAs showed opposite expression patterns in response to *B. japonicum* infection (Figure 1); gma-miR171o expression was suppressed upon bacterial infection, while gma-miR171q was induced.

### Gma-miR171o and Gma-miR171q Negatively Regulate Soybean Nodulation

**FIGURE 2:**
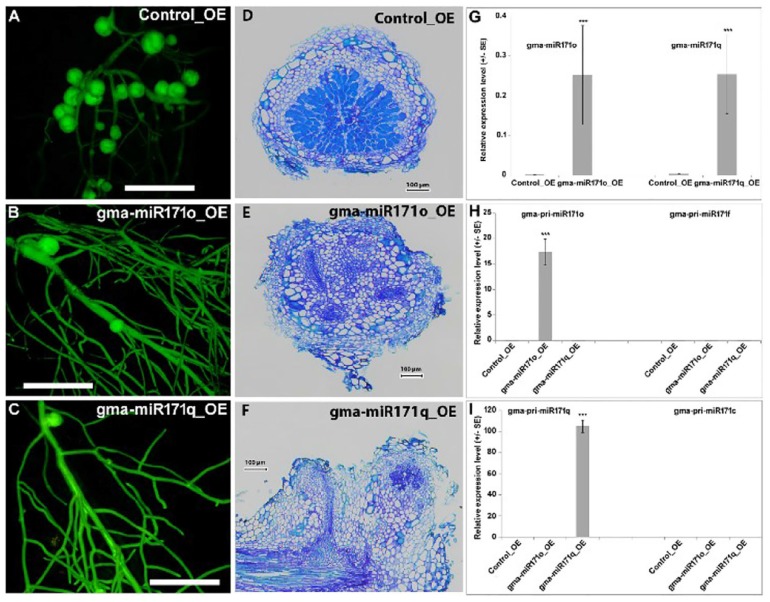
**(A–I)** Overexpression of gma-miR171o and gma-miR171q inhibits soybean nodulation.

In order to understand the functional relevance of gma-miR171o and gma-miR171q, each miRNA gene was ectopically expressed in soybean hairy roots from the strong, constitutive CvMV promoter (Li et al., 2010). In both cases, transgenic roots expressing the miRNA genes showed a significant reduction in nodule formation 4 weeks post-inoculation with *B. japonicum* (Figure 2A–C and Supplementary Figure S2), which indicated that both gma-miR171o and gma-miR171q negatively affect soybean nodulation. Ectopic expression of these two miRNAs formed very few, small and white nodules in soybean hairy root transgenic tissues (Figure 2A–C and Supplementary Figure S2). Microscopic analysis revealed that these white nodules were unable to accommodate bacterial invasion in the nodule cortical cells, suggesting a defect in both infection and subsequent nitrogen fixation (Figure 2D–F).

To validate this phenotype, we conducted stem-loop qRT-PCR to quantify the mature miRNA levels (Yan et al., 2015). Both gma-miR171o and gma-miR171q levels increased significantly in the transgenic roots, as compared to a vector control (Figure 2G). Thus, both miRNAs were overexpressed at similar levels. Given that both primary miRNA171 transcripts give rise to identical miRNAs, it is not surprising that the two constructs also give an identical nodulation phenotype. To confirm miRNA abundances, we designed precursor-specific primers (Supplementary Table S1) and the resulting qRT-PCR data clearly indicated that both miRNA precursors were activated during ectopic expression (Figure 2H,I). Given that soybean has 21 miR171 genes, we also sought to eliminate the possibility that the expression of one or more of these genes could be responsible for the nodulation phenotype. Hence, we also designed primers to the two closest homologs (miR171c and miR171f) (Supplementary Figure S1A). The expression level of these genes was not changed in the transgenic roots (Figure 2H,I).

### NSP2 and SCL6 Are Targets of miRNA171

**FIGURE 3:**
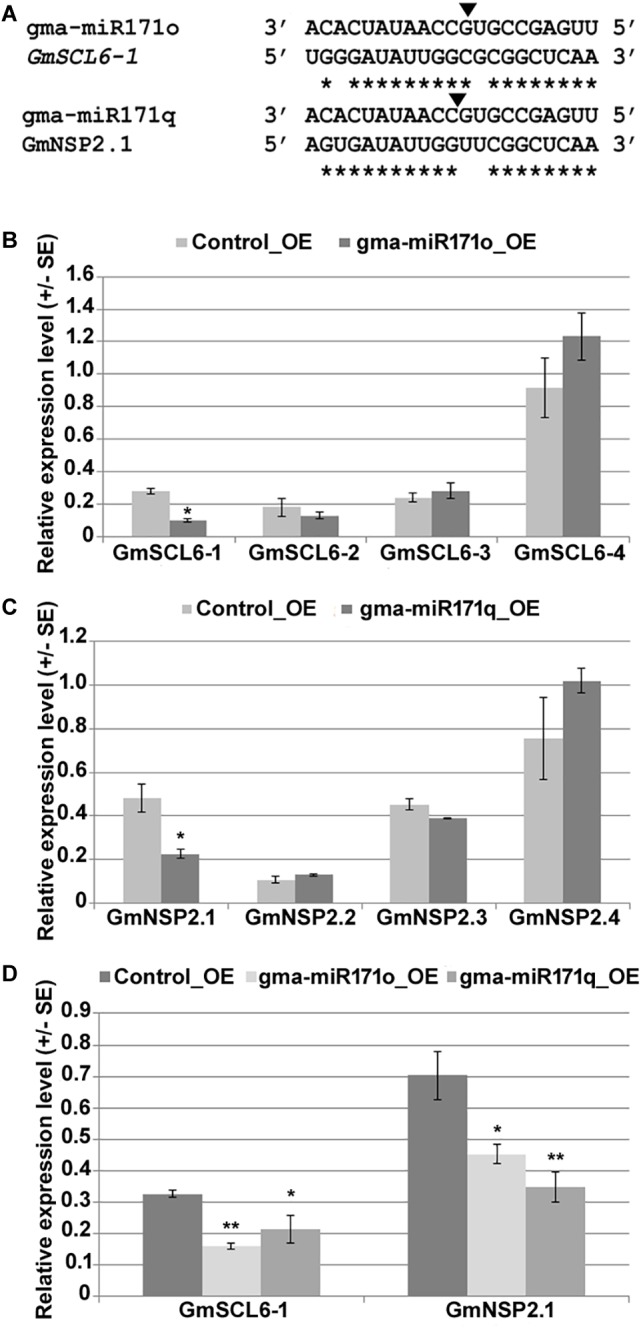
*GRAS* family genes *GmSCL-6-1* and *GmNSP2* are targets of gma-miR171o and gma-miR171q. **(A)** Alignment of mature micro-RNA sequences of gma-miR171o and gma-miR171q with their corresponding target genes *GmSCL6-1* and *GmNSP2* in soybean. Black arrowhead indicates the position of cleavage target site, ^∗^marks at the bottom indicate the nucleotide complementarity between miR171 copies with their corresponding targets. **(B,C)** qRT-PCR expression analysis of target gene and their closest homologs in overexpression tissues of gma-miR171o **(B)** and gma-miR171q **(C)** in soybean transgenic hairy roots. **(D)**, Confirmation of both target genes, *GmSCL6-1* and *GmNSP2.1* expression in gma-miR171o and gma-miR171q overexpression tissues. ^∗^ and ^∗∗^ in **(B–D)** indicates *t*-test significance at *p* < 0.05 and *p* < 0.01, respectively.

Parallel Analysis of RNA Ends analysis reveals the potential targets of each miRNA, which provides valuable information to understand and explore the function of specific miRNAs (Yan et al., 2015). Utilizing our nodulation PARE data as previously published (Yan et al., 2015), we confirmed that in soybean two GRAS family transcription factors (TFs), namely, *Scarecrow like-6* and *Nodulation-signaling pathway 2* are the likely target genes of gma-miR171o and gma-miR171q (Figure 3A). Based on phylogenetic analysis with two other model legumes, *M. truncatula* and *L. japonicus*, and a non-legume species, *Arabidopsis*, we re-named these targets as *GmSCL6-1* and *GmNSP2.1* (Supplementary Figures S3A,B). The NSP2 TF is well documented as a critical member of the nodulation signaling pathway (Kalo et al., 2005; Murakami et al., 2006; Hofferek et al., 2014), while a functional role for the SCL6 in nodulation has not been reported, although this TF has been studied in *Arabidopsis* (Pysh et al., 1999). In order to confirm *GmSCL6-1* and *GmNSP2.1* as targets for gma-miR171o and gma-miR171q we measured the expression level of each gene, as well as their closest homologs, in hairy roots ectopically expressing each of the miRNAs (Figure 3B,C). As expected, we found that the transcript levels of only *GmSCL6-1* and *GmNSP2.1* were significantly reduced in the transgenic roots, while the expression levels of the homologs were unaffected (Figure 3B,C). Since the mature miRNA sequences of these two miRNAs are identical, we also assessed whether expression of *GmSCL6-1* and *GmNSP2.1* mRNA was affected in transgenic roots expressing the opposite miRNA precursor. Interestingly, consistent with the identical nature of the miRNA, we found that both miRNAs could target either *GmSCL6-1* or *GmNSP2.1* transcript (Figure 3D). These data support the hypothesis that gma-miR171o and gma-miR171q might regulate the abundance of *GmSCL6-1* and/or GmNSP*2.1* mRNA level to restrict soybean nodulation.

**FIGURE 4:**
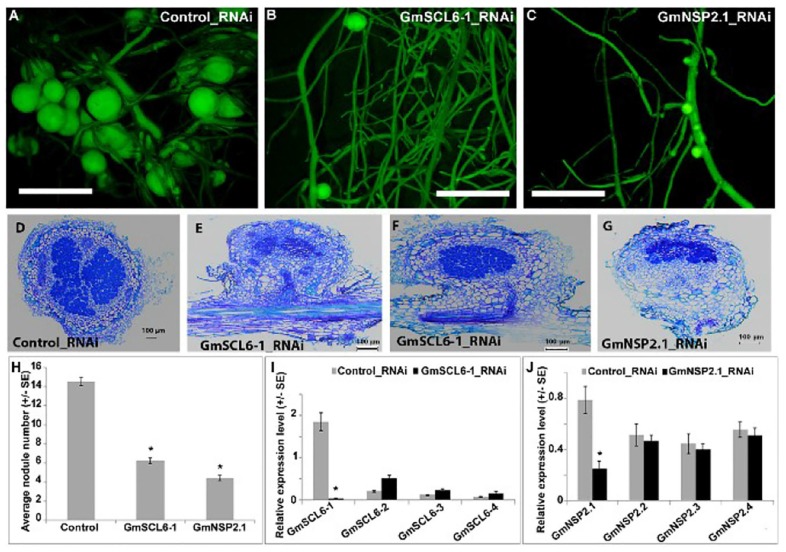
**(A–J)** *GmSCL6-1* and *GmNSP2.1* are required for soybean nodulation.

Given that NSP2 has a well-documented role in nodulation, one hypothesis is that gma-miR171 controls nodulation by exclusively targeting this gene. To test this possibility, we used RNAi to specifically silence the expression of *GmSCL6-1* or *GmNSP2.1* in transgenic hairy roots (Figure 4). For efficient silencing of these two genes, we designed RNAi constructs to target the 3′ UTR regions. Transgenic roots silenced for either *GmSCL6-1* or *GmNSP2.1* expression showed a significant reduction (approximately 60 and 70%) in nodule formation, respectively (Figure 4A–C,H). Microscopic analysis of the nodules from the silenced roots revealed an almost similar nodule structural phenotype, except that in rare instances some nodules formed on *GmSCL6-1* RNAi transgenic roots had infected cells identical to the wild type (Figure 4D–G). To confirm that the observed phenotypes were caused solely by *GmSCL6-1* and *GmNSP2.1* silencing, and not that of their paralogs, we quantified the expression of paralogs closest to *GmSCL6-1* and *GmNSP2.1*, for the RNAi transgenic roots and nodules. Only *GmSCL6-1* and *GmNSP2.1* transcript levels were significantly repressed by RNAi (Figure 4I,J). Altogether, these data suggest that gma-miR171o and gma-miR171q function in nodulation by targeting both *GmSCL6-1* and *GmNSP2.1* gene expression.

### Gene Expression and Promoter Localization of *GmSCL6-1* and *GmNSP2.*1 Are Inversely Correlated With Gma-miR171o and Gma-miR171q

**FIGURE 5:**
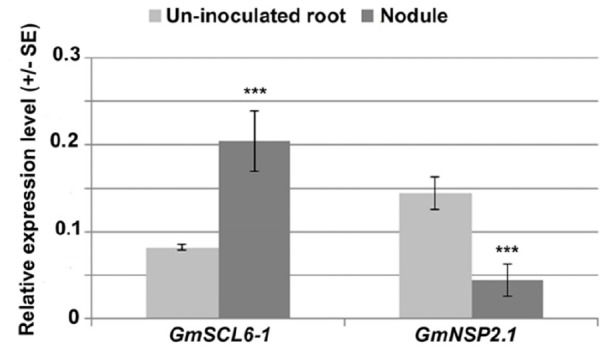
Expression analysis of target genes, *GmSCL6-1* and *GmNSP2.1* in soybean hairy root transgenic tissues.

One explanation for these results, given the identical sequence of gma-miR171o and gma-miR171q, is that these two miRNAs target *GmSCL6-1* and *GmNSP2.1* expression in distinct tissues or at distinct temporal stages of nodulation. To explore this hypothesis from the standpoint of the target genes, we examined the patterns of *GmSCL6-1* and *GmNSP2.1* mRNA expression as measured by qRT-PCR (Figure 5). The expression patterns of the target genes were inversely correlated with the abundance of their corresponding miR171. For example, gma-miR171o expression decreased significantly in nodules compared to uninfected roots (Figure 1) while its target gene *GmSCL6-1* mRNA level increased in nodules in response to bacterial infection (Figure 5). On the other hand, gma-miR171q was induced significantly in nodules upon *B. japonicum* infection (Figure 1) while its target gene *GmNSP2.1* transcript level was suppressed (Figure 5). The expression patterns of *GmSCL6-1* and *GmNSP2.1* were consistent and correlated with our earlier RNA-seq data available in the SoyKB database^2^ (Supplementary Figure S4).

### miR171o and miR171q Are Critical for NSP2 and SCL6 Regulation During Soybean Nodulation

**FIGURE 6:**
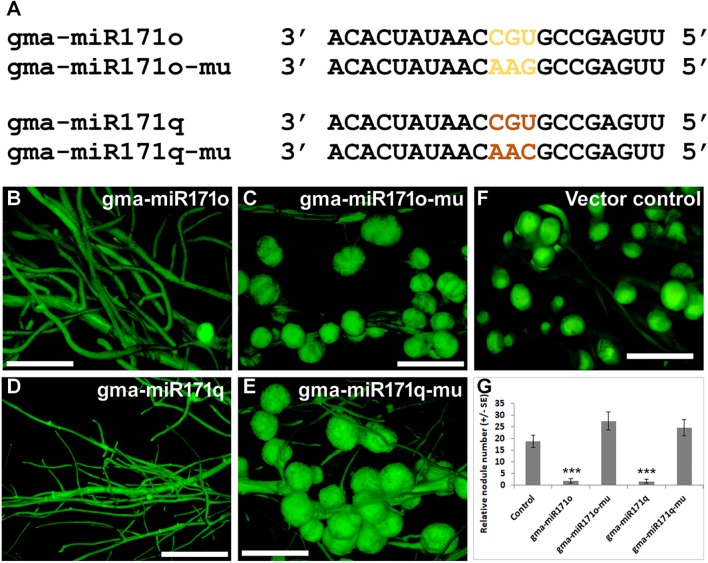
Key nucleotides at mature microRNA sequences of miR171o and -q are critical for nodulation in soybean. **(A)** Mature miRNA sequences of gma-miR171o and gma-miR171q and the positions of 3 nt mutation (highlighted) that are critical for potential mRNA cleavage. **(B–E)** Root nodule phenotypes on soybean transgenic hairy roots in the overexpression constructs of gma-miR171o **(B)**, gma-miR171o-mu **(C)**, gma-miR171q **(D)**, and gma-miR171q-mu **(E)** with/without mutation in the potential mRNA cleavage sites. “mu” indicates the mutated version of the precursor overexpression construct. **(F)** Root nodule phenotype of positive vector control in soybean transgenic hairy roots. **(G)** Quantification of relative nodule numbers in the overexpression constructs of gma-miR171o and gma-miR171q with/without mutation in the potential mRNA cleavage sites that formed on transgenic hairy roots of soybean. Error bar represents ±SE and ^∗∗∗^mark indicate *t*-test significance at *P* < 0.001.

**FIGURE 7:**
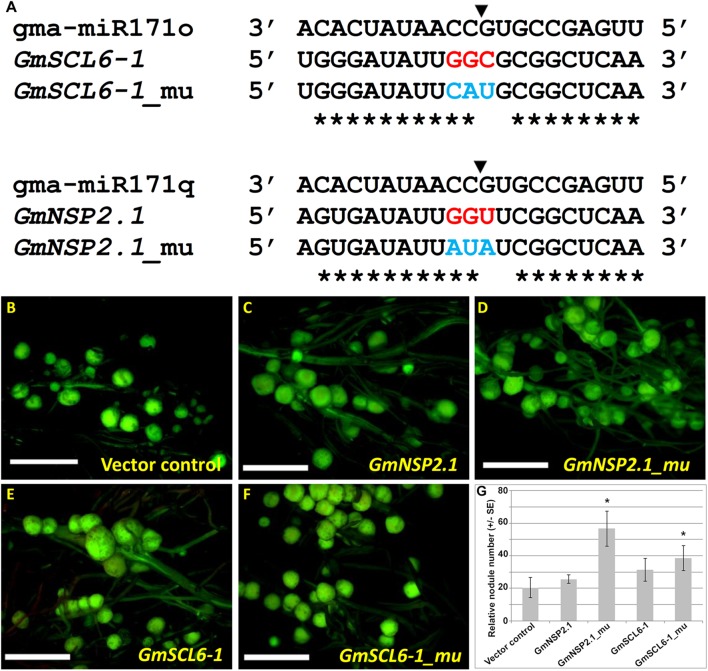
Nodulation phenotype of *GmSCL6-1* and *GmNSP2.1* overexpression in soybean transgenic hairy roots. **(A)** Alignment of mature microRNA sequences of gma-miR171o and gma-miR171q with *GmSCL6-1* and *GmNSP2.1* target gene with or without mutation at the key target cleavage positions. The black arrowhead indicates the cleavage position and the blue color-coded letter indicate target mutation site for *GmSCL6-1* and *GmNSP2.1*, respectively. The asterisks indicate nucleotide complementarity between gma-miR171o and gma-miR171q with their corresponding targets. **(B–F)** Images of the nodulation phenotype observed in the overexpression constructs of vector control **(B)**, *GmNSP2.1* **(C)**, *GmNSP2.1*_mu **(D)**, *GmSCL6-1* **(E)**, and *GmSCL6-1*_mu **(F)**. “mu” indicates the mutated version of overexpression constructs for target genes, *GmNSP2.1* and *GmSCL6-1*, respectively. **(G)** Relative nodulation counts on each transgenic hairy root from overexpression constructs shown in panels **(B–F)**. Error bar represents ±SE and ^∗^ mark indicate *t*-test significance at *P* < 0.05.

As noted before, our hypothesis is that gma-miR171o and gma-miR171q target the mRNA of *GmSCL6-1* and *GmNSP2.1* for cleavage in distinct root tissues, thereby controlling distinct steps in soybean nodulation. To provide a further test of this hypothesis, specific mutations were made in gma-miR171o and gma-miR171q to disrupt their ability to cleave mRNA. It was previously shown that key base pair positions (i.e., nucleotides 9 to 12) in a mature miRNA sequences are crucial for target cleavage activity (Kasschau et al., 2003). Mutations were made in the corresponding positions of both gma-miR171o and-q, and in the sequence of *GmNSP2.1* and *GmSCL6-1* genes to reduce the cleavage of their mRNAs (Figure 6, 7). Transgenic roots expressing mutated versions of either gma-miR171o or gma-miR171q, lacking the ability to cleave mRNA, resulted in normal nodulation of soybean roots (Figure 6C,E,G), a result distinct from that found when wild-type versions of these miRNAs were ectopically expressed in transgenic roots (Figure 6B,D,G). More interestingly when transgenic roots expressed mutated versions of either *GmSCL6-1* or *GmNSP2.1*, lacking the ability to be cleaved by either gma-miR171o or gma-miR171q, they formed a significantly (*P* < 0.05) higher number of nodules compared to transgenic roots ectopically expressing wild-type *GmSCL6-1* and *GmNSP2.1* (Figure 7B–G). This indicates that microRNA regulation plays a crucial role in restricting expression domains and thus spatial action of these genes. Furthermore, these results are consistent with the notion that it is the specific targeting of the *GmSCL6-1* and *GmNSP2.1* mRNA by gma-miR171o or gma-miR171q that is crucial for the functional role of these miRNA in controlling soybean nodulation.

### Gma-miR171o and Gma-miR171q Target GmSCL6-1 and GmNSP2.1 *in planta*

**FIGURE 8:**
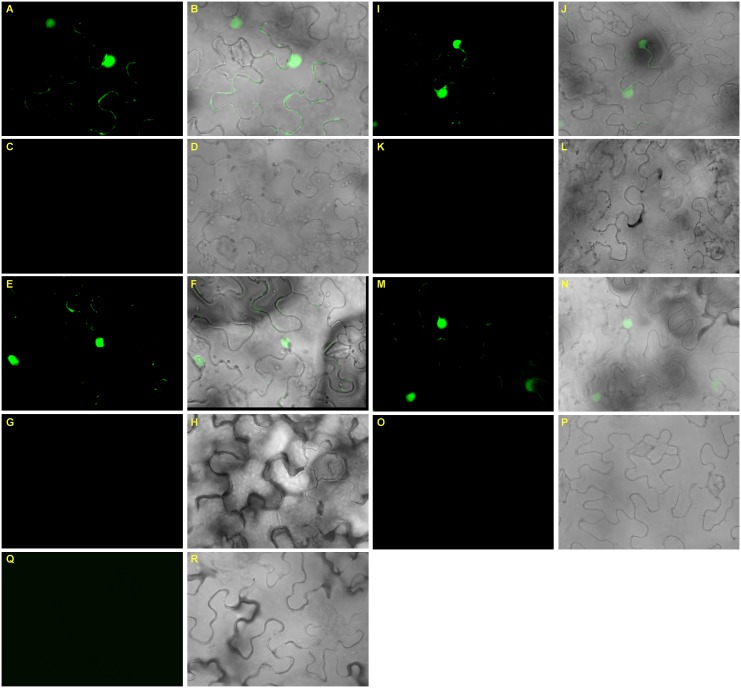
Transient co-expression analysis of gma-miR171o and gma-miR171q miRNA precursors with their corresponding target genes in *N. benthamiana* confirms cleavage activity. **(A–D)** Interaction of gma-miR171o with *GmNSP2.1*, either the mutated version of gma-miR171o **(A,B)** or non-mutated **(C,D)** and interaction of gma-miR171o with *GmSCL6-1*, either the mutated version of gma-miR171o **(E,F)** or non-mutated **(G,H)**. **(I–L)** Interaction of gma-miR171q with *GmNSP2.1*, either the mutated version of gma-miR171q **(I,J)** or non-mutated **(K,L)** and the interaction of gma-miR171q with *GmSCL6-1*, either the mutated version of gma-miR171q **(M,N)** or non-mutated **(O,P)**. Note, panels in 2nd and 3rd rows are corresponding images from 1st to 4th rows. **(Q,R)** Indicates images of a vector control.

To understand the complexity of miRNA-target interaction and their direct cleavage activity *in planta*, we transiently expressed gma-miR171o and gma-miR171q and their target genes, *GmSCL6-1* and *GmNSP2.1* in *N. benthamiana* leaves. We transformed both mutated and non-mutated versions of gma-miR171o and gma-miR171q, and also *GmSCL6-1* and *GmNSP2.1* fused in-frame with *GFP* (see Materials and Methods) into *Agrobacterium* GV3101 cells. These constructs were then co-infiltrated into *N. benthamiana* leaves (Figure 8). The results show that when either the mutated version of gma-miR171o or gma-miR171q was co-infiltrated with *GmSCL6-1* or *GmNSP2.1*, strong GFP expression was found in the nucleus, consistent with the role of these nuclear localized proteins (data not shown) as transcription factors (Figure 8A,C,E,G,I,K,M,O). However, when the active versions of gma-miR171o or gma-miR171q were used, no GFP signal was detected, suggesting miRNA-mediated cleavage of the target genes (*GmSCL6-1* or *GmNSP2.*1) fused to GFP (Figure 8B,D,F,H,J,L,N,P).

### Overexpression of miRNA171o/q Leads to Suppression of Early Nodulin Genes

**FIGURE 9:**
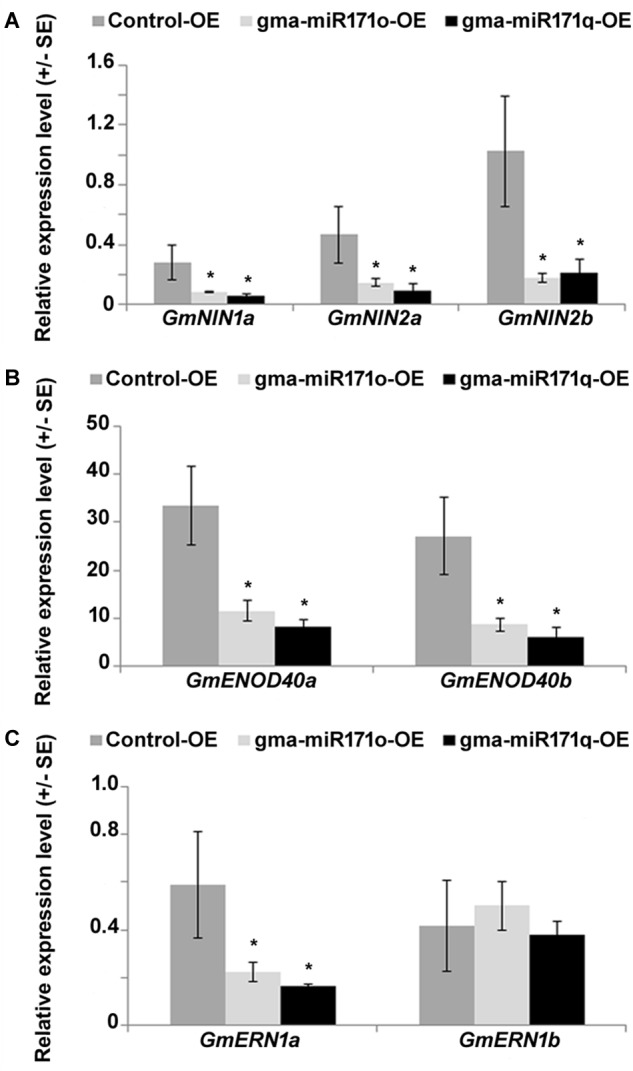
Suppression of early nodulin transcripts in gma-miR171o and gma-miR171q overexpression tissues, measured using transgenic soybean hairy roots and nodules after 4 weeks of *B. japonicum* inoculation. qRT-PCR analysis of early nodulin genes, *GmNIN1a*, *GmNIN2a*, and *GmNIN2b* **(A)**, *GmENOD40a* and *GmENOD40b* **(B)**, and *GmERN1a* and *GmERN1b* **(C)**. Three independent biological replicates from each overexpression line were used for qRT-PCR analysis. Error bars represents ±SE and asterisks indicate *t*-test significance at a *P*-value < 0.05. “OE” indicates overexpression.

Studies in *L. japonicus* and *M. truncatula* demonstrated the role of NSP2 in regulating the expression of key genes in nodule formation (Oldroyd and Long, 2003; Kalo et al., 2005; Heckmann et al., 2006; Murakami et al., 2013). Among those genes whose expression is affected are *NIN*, *ENOD40*, and *ERN* (Hayashi et al., 2012). We measured the expression of the soybean homologs of these genes to test the effect of down regulation of *GmNSP2.1* or *GmSCL6.1* (Figure 9). As measured by qRT-PCR, with the exception of *GmERN1b*, the expression of these transcripts was significantly reduced in transgenic soybean roots ectopically expressing either gma-miR171o or gma-miR171q (Figure 9), suggesting that gma-miR171o and-q mediated regulation of *GmSCL6-1* and *GmNSP2.1* might influence fine tuning of early nodulin gene expression in order to suppress soybean nodulation.

## Discussion

Over the past decade, the regulatory roles of miRNAs have been established in a variety of pathways in both animals and plants. This includes roles in regulating steps of legume nodulation. However, to date, there are few examples in which the details of miRNA function have been clarified beyond showing differential expression of a specific miRNA during rhizobial infection (De Luis et al., 2012; Yan et al., 2013; Hofferek et al., 2014; Wang et al., 2014). The aim of our study was to decode the function of two members of the miR171 family in soybean, building on work documenting the role of miR171 family members in the model legumes *L. japonicus* and *M. truncatula* (De Luis et al., 2012; Hofferek et al., 2014). gma-miR171o and gma-miR171q were of interest since they had identical mature sequences, but highly variable stem loop sequences, consistent with functional divergence beyond their target homology. The data presented are consistent with a model by which the miRNA genes are expressed in a spatially unique pattern, which allows the corresponding miRNA species to target unique genes, *GmNSP2.1* or *GmSCL6.1*, and regulate crucial but different steps in the nodulation process. Thus, in addition to GmNSP2, whose function could be inferred from previous studies, the results of our study also implicate GmSCL6 as a novel, important regulator of soybean nodulation. The distinct regulators controlled by these two similar transcription factors remain to be elucidated.

Since soybean is a partially diploidized tetraploid (Shultz et al., 2006), it explains the existence of a large number of gene copies in the genome. In the case of miR171, there are 21 predicted family members. Consistent with our previous publication that looked at gene duplication at a genome level (Roulin et al., 2013), miRNA gene duplication allowed for sub-functionalization of gene function, exemplified by unique targeting of *GRAS* family genes by the two miR171 family members. We found that gma-miR171o and gma-miR171q have highly divergent precursor structures and sequences, yet their 21 nt, mature microRNA sequences are identical. We examined whether these identical miRNAs target the same gene and found that the functions of gma-miR171o and gma-miR171q are highly dependent on their spatiotemporal regulation. This includes restriction to the epidermal cell layer or site of lateral root emergence site. In response to *B. japonicum* inoculation, promoter activity shifts, possibly to optimize microRNA activity. Based on PARE analysis and our validation experiments, these two miRNAs target two distinct genes.

It was earlier reported that miR171 acts as a negative regulator for legume-nodulation, as well as for the mycorrhizal symbiosis (De Luis et al., 2012; Lauressergues et al., 2012). In soybean, we also confirmed that miR171 negatively controlled nodulation by targeting transcripts of GRAS TFs. Specifically, ectopic expression of gma-miR171o and gma-miR171q miRNA genes significantly inhibited nodulation with the formation of occasional nodules that are defective in nitrogen fixation. In *L. japonicus*, miR171 controls *NSP2* to regulate bacterial infection in nodules (De Luis et al., 2012). That work showed that miR171a and miR171c were abundant in mature nodules; one of which (miR171c) was highly expressed in nodules. Bacterial infection was needed to induce miR171c expression in nodules as spontaneous nodules without bacteria do not express the miRNA (De Luis et al., 2012). Similarly, in *Medicago*, cleavage of *MtNSP2* is directed by miR171h and required for both nodule formation and mycorrhizal signaling pathway (Branscheid et al., 2011; Devers et al., 2011; Lauressergues et al., 2012). In Medicago, cytokinin post-transcriptionally regulates MtNSP2 via miR171h-NSP2 silencing during nodule primordia formation (Ariel et al., 2012). We demonstrate that gma-miR171o is highly expressed at the root epidermis in uninfected roots and repressed during nodule formation after *B. japonicum* inoculation. In contrast to gma-miR171o, in uninfected roots, gma-miR171q is active in the sub-epidermal layer of cells where lateral roots emerge and it accumulates in nodules. This contrasting pattern of accumulation in both transcripts and promoter activity led us to further characterize these miRNAs. Using PARE analysis from our prior work (Arikit et al., 2014; Yan et al., 2015), we confirmed that *GmSCL6-1* and *GmNSP2.1* are targeted by gma-miR171o and gma-miR171q; these are required for functional nodulation. Using transient *in planta* expression, together with other results, we confirmed that gma-miR17o and gma-miR171q directly cleaved their *GmSCL6-1* and *GmNSP2.1* target. We were also able to localize promoter activity as well as transcript expression of both gma-miR171o and gma-miR171q, and their targets, in an anti-correlated pattern.

*NODULATION SIGNALING PATHWAY 2* has not been well characterized in soybean despite substantial work in *L. japonicus* and *M. truncatula* (Oldroyd and Long, 2003; Kalo et al., 2005; Murakami et al., 2006; Hirsch et al., 2009). Mutation of *NSP2* genes in these two species affects root hair curling in response to Nod factor release from rhizobia bacteria. The *nsp2* mutants are impaired in infection and cortical cell division upon *S. meliloti* inoculation, displaying no nodule phenotype (Heckmann et al., 2006). In the nod factor signaling pathway, *NSP2* acts downstream of the calcium-spiking response. In *M. truncatula*, it was reported that NSP1 and NSP2 form a heterodimer that is required for the association with the *ENOD11* promoter upon Nod factor elicitation (Hirsch and Oldroyd, 2009). In our work, we characterized the molecular function of *GmNSP2.1* and *GmSCL6-1* in soybean, demonstrating that *GmSCL6-1* is a GRAS family TF that functions in the Nod factor signaling.

Scarecrow (SCR) transcription factor is one of the eight subfamilies of the GRAS proteins. In *Arabidopsis*, SCR acts downstream of SHORT-ROOT to regulate shoot and root radial patterning (Helariutta et al., 2000). In addition, *Arabidopsis* SCL3 directly interacts with the Gibberellin (GA) repressor, namely DELLA proteins. SCL3 is the positive regulator of GA signaling and acts immediately downstream of DELLA (Zhang et al., 2011). In *Arabidopsis*, miR171c targets SCL6-II, SCL6-III, and SCL6-IV to regulate shoot branching (Wang et al., 2010). Although other members of the GRAS family, including NSP1 and NSP2, have been shown to play important roles in legume-rhizobium symbiosis (Hirsch and Oldroyd, 2009), direct evidence of a role for SCR or SCL protein function in rhizobium-legume symbiosis has not been reported. Our work shows that *GmSCL6-1* is the target of gma-miR171o and acts as a negative regulator of soybean nodulation in response to *B. japonicum* inoculation. This further confirms the important role of miR171/SCL6 interaction as the regulator of different plant processes and especially the rhizobium symbiosis.

Nodulins are class of marker genes in legumes that are activated during legume-rhizobium symbiosis (Nguyen et al., 1985; Franssen et al., 1987; Weaver et al., 1994; Fang and Hirsch, 1998; Gronlund et al., 2005; Vernie et al., 2015; Kawaharada et al., 2017). In soybean, it was shown that miR172c targets the transcription factor *NNC1* which binds to the ENOD40 promoter, an early nodulin that control transcriptional activity for nodulation signaling (Wang et al., 2014). Based on this study, we looked at the expression patterns of three different types of early nodulin genes (*NIN*, *ENOD40*, and *ERN*) in the overexpression tissues of gma-miR171o and gma-miR171q. Surprisingly, except *GmERN1b*, all the selected early nodulin genes suppressed their transcript level significantly in miR171 overexpression tissues, underscoring the important role of miRNAs as gene regulators.

Altogether, the experimental evidence presented indicate that the cellular levels of microRNA are precisely controlled for optimal spatiotemporal regulation of target genes, adding an additional layer of complexity to the signaling processes that mediated root nodule formation.

## Author Contributions

MH designed the experiments. MH and NH performed the research and analyzed the data. ZY, KT, and BM revised the manuscript. MdH wrote the manuscript with input from NH and GS.

## Conflict of Interest Statement

The authors declare that the research was conducted in the absence of any commercial or financial relationships that could be construed as a potential conflict of interest.

## Footnotes

1 www.mirbase.org

2 soykb.org

## Supplementary Material

Figure S1 Features of the miR171 family in soybean. **(A)** Phylogenetic relationship of 21 primary transcripts of gma-miR171 family members. **(B)** Alignment of primary transcript sequences of gma-miR171o and gma-miR171q. Green color represents alignment of the two mature miRNAs, gma-miR171o, and gma-miR171q.

Figure S2 Average nodule number formed on transgenic hairy roots at 4 wpi by ectopic expression of gma-pri-miR171o and gma-pri-miR171q. Nodule number was counted from at least 20 transgenic hairy roots from two independent experiments. ^∗∗∗^indicate *t*-test significance of *P* < 0.0005.

Figure S3 Soybean contains multiple copies of GmNSP2 and GmSCL6 proteins and they are phylogenetically distinct member of GRAS super family proteins. **(A)** Phylogenetic tree of GmNSP2 and GmSCL6 and their homologs in *Arabidopsis*, *L. japonicus* and *M. truncatula*. **(B)** Phylogenetic relationship of soybean homologs of GmNSP1, GmNSP2, and GmSCL6 along with proteins from *L. japonicus* and *M. truncatula*.

Figure S4 Expression patterns of *GmNSP2.1* and *GmSCL6-1* in early stage of soybean root hair infection and the later stage of nodules after *B. japonicum* inoculation. RPKM values from different time points as indicated below were collected from the SoyKB database (soykb.org).

Table S1 Primer used for this study.
