# Corrigendum: Characterization of the Spatial and Temporal Expression of Two Soybean miRNAs Identifies SCL6 as a Novel Regulator of Soybean Nodulation

**DOI:** 10.3389/fpls.2019.01692

**Published:** 2020-02-12

**Authors:** Md Shakhawat Hossain, Nhung T. Hoang, Zhe Yan, Katalin Tóth, Blake C. Meyers, Gary Stacey

**Affiliations:** ^1^C.S. Bond Life Science Center, Divisions of Plant Sciences and Biochemistry, University of Missouri, Columbia, MO, United States; ^2^Donald Danforth Plant Science Center, St. Louis, MO, United States

**Keywords:** miRNA, miR171, GRAS TF, *Scarecrow like-6*, NSP2, nodulation, symbiosis, soybean

It was brought to our attention that, in the original article, inappropriate manipulation of **Figures 1** and **5** took place, specifically, **Figures 1B, 1C, 1D, 1E, 1F, 1G** and **5B, 5C, 5D, 5E, 5F, 5G**. Upon review, we have agreed that these figures be removed from the article. The manipulation of the figures involved an effort to increase the contrast of the images to make them more presentable.

Although this did not change the interpretation of the data, the manipulation was clearly against our lab policy and the policies of the journal. Given that other data in the paper (e.g., qPCR and other experiments) confirm the data presented in these figures, we feel that the conclusions of the paper remain valid even with the figures removed.

To avoid any additional questions or concerns, we have also corrected **Figures 2D, 2E** and **2F**, as well as **Figures 4D, 4E, 4F**, and **4G**, and replaced these figures with original images. The corrected figures appear below. Due to the changes mentioned above, a correction has been made to the **Results**, subsection **Gma-miR171o and Gma-miR171q Exhibit Distinct Expression Patterns in Response to Bacterial Infection**:

“To understand the symbiotic role of gma-miR171o and gma-miR171q in soybean, we measured the relative expression level of these two miRNAs in nodules 3 weeks post-inoculation with *B. japonicum*, as compared to uninfected root tissues (**Figure 1A**). Interestingly, these two miRNAs showed opposite expression patterns in response to *B. japonicum* infection (**Figure 1A**); gma-miR171o expression was suppressed upon bacterial infection, while gma-miR171q was induced.”

A correction has also been made to the **Results**, subsection **Gene Expression and Promoter Localization of *GmSCL6-1* and *GmNSP2*.1 Are Inversely Correlated With Gma-miR171o and Gma-miR171q**. Paragraph two has been removed entirely.

**Figure 1:**
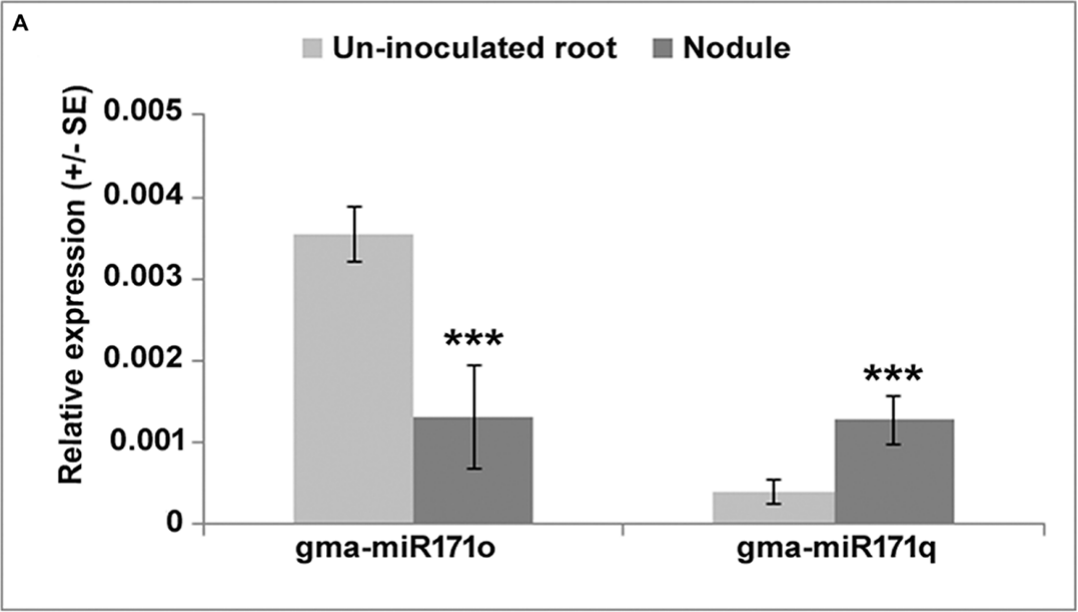
Expression patterns of gma-miR171o and gma-miR171q during symbiosis with *B. japonicum*.

**Figure 2:**
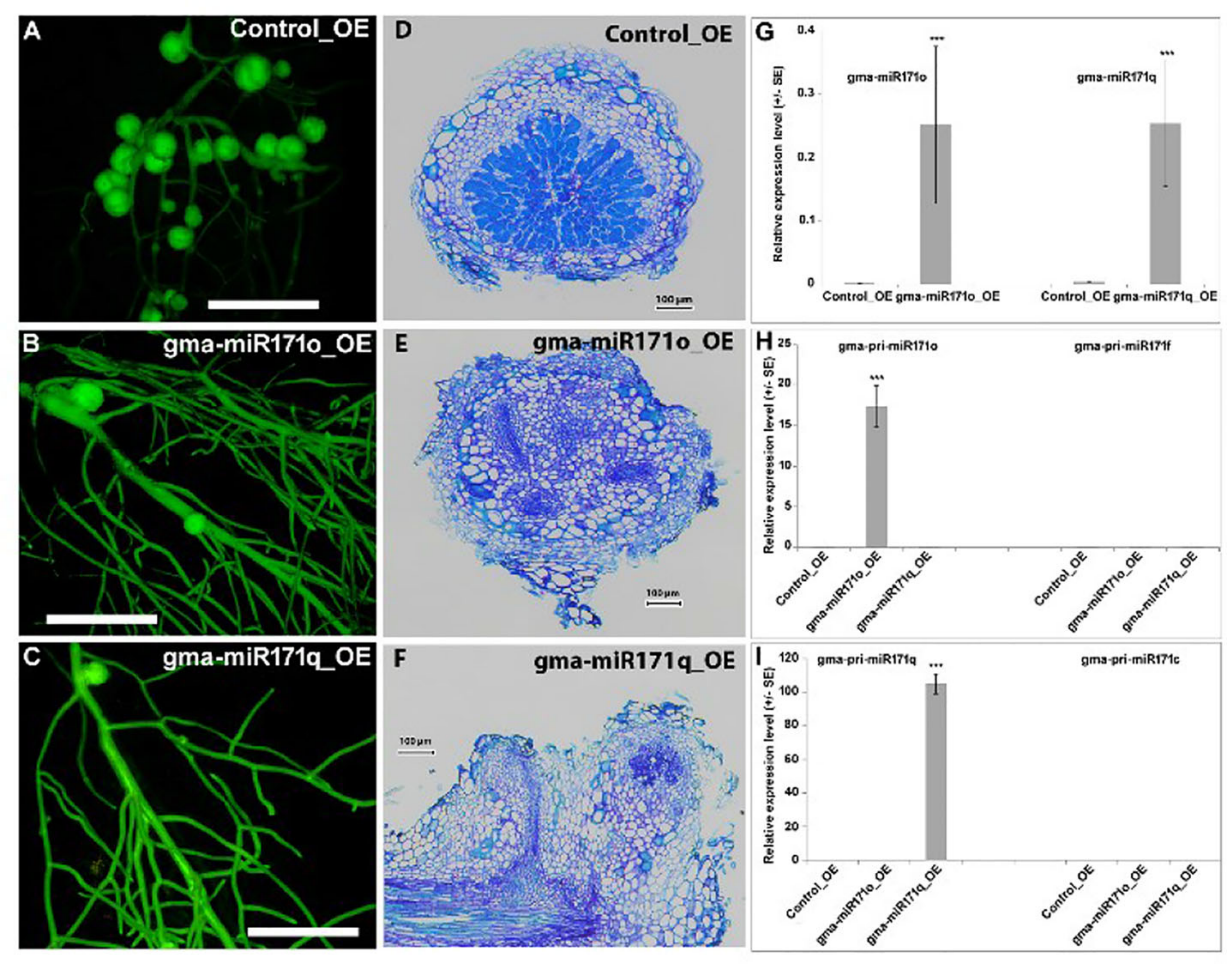
Overexpression of gma-miR171o and gma-miR171q inhibits soybean nodulation.

**Figure 4:**
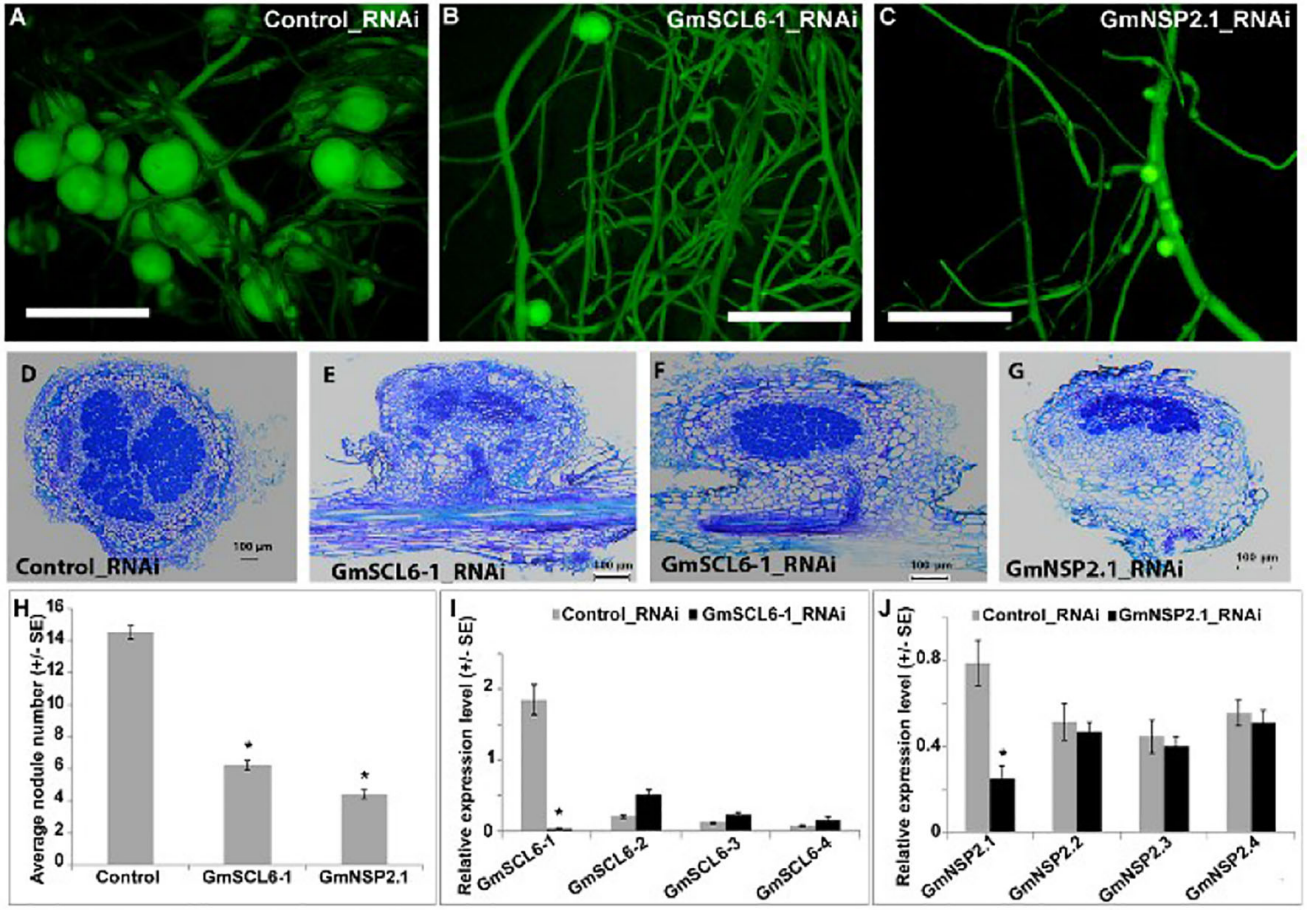
*GmSCL6-1* and *GmNSP2.1* are required for soybean nodulation.

**Figure 5:**
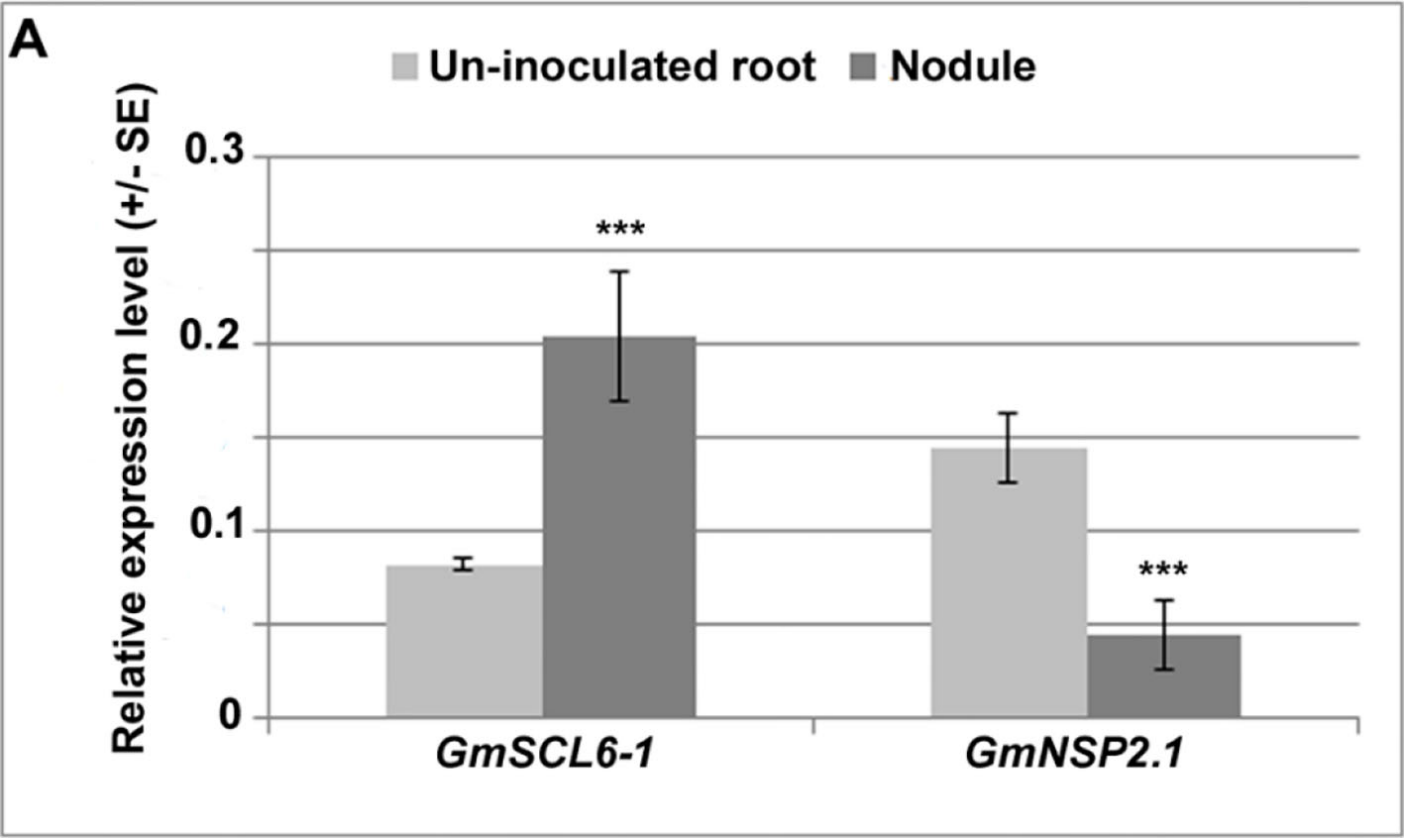
Expression analysis of target genes, *GmSCL6-1* and *GmNSP2.1* in soybean hairy root transgenic tissues.

The authors apologize for these errors and state that they do not change the scientific conclusions of the article in any way. The original article has been updated.

