# Supplementary figures and images for: Characterization of the Spatial and Temporal Expression of Two Soybean miRNAs Identifies SCL6 as a Novel Regulator of Soybean Nodulation
Source: Front Plant Sci. 2019 Apr 16;10:475. doi: 10.3389/fpls.2019.00475 (PMC6477095; doi:10.3389/fpls.2019.00475)

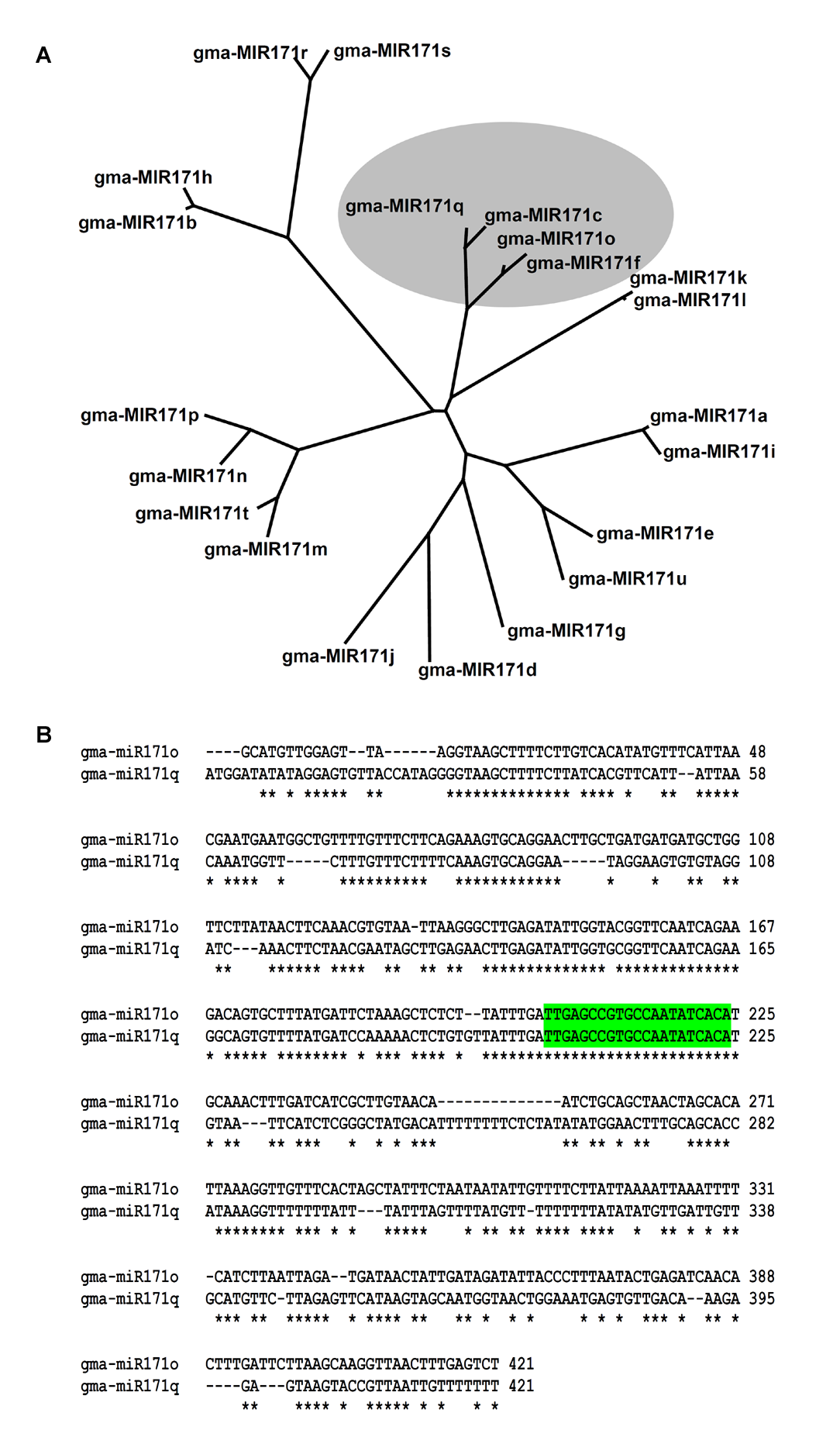

Supplement: Figure S1 — Features of the miR171 family in soybean. (A) Phylogenetic relationship of 21 primary transcripts of gma-miR171 family members. (B) Alignment of primary transcript sequences of gma-miR171o and gma-miR171q. Green color represents alignment of the two mature miRNAs, gma-miR171o, and gma-miR171q. [file Image_1.TIF]

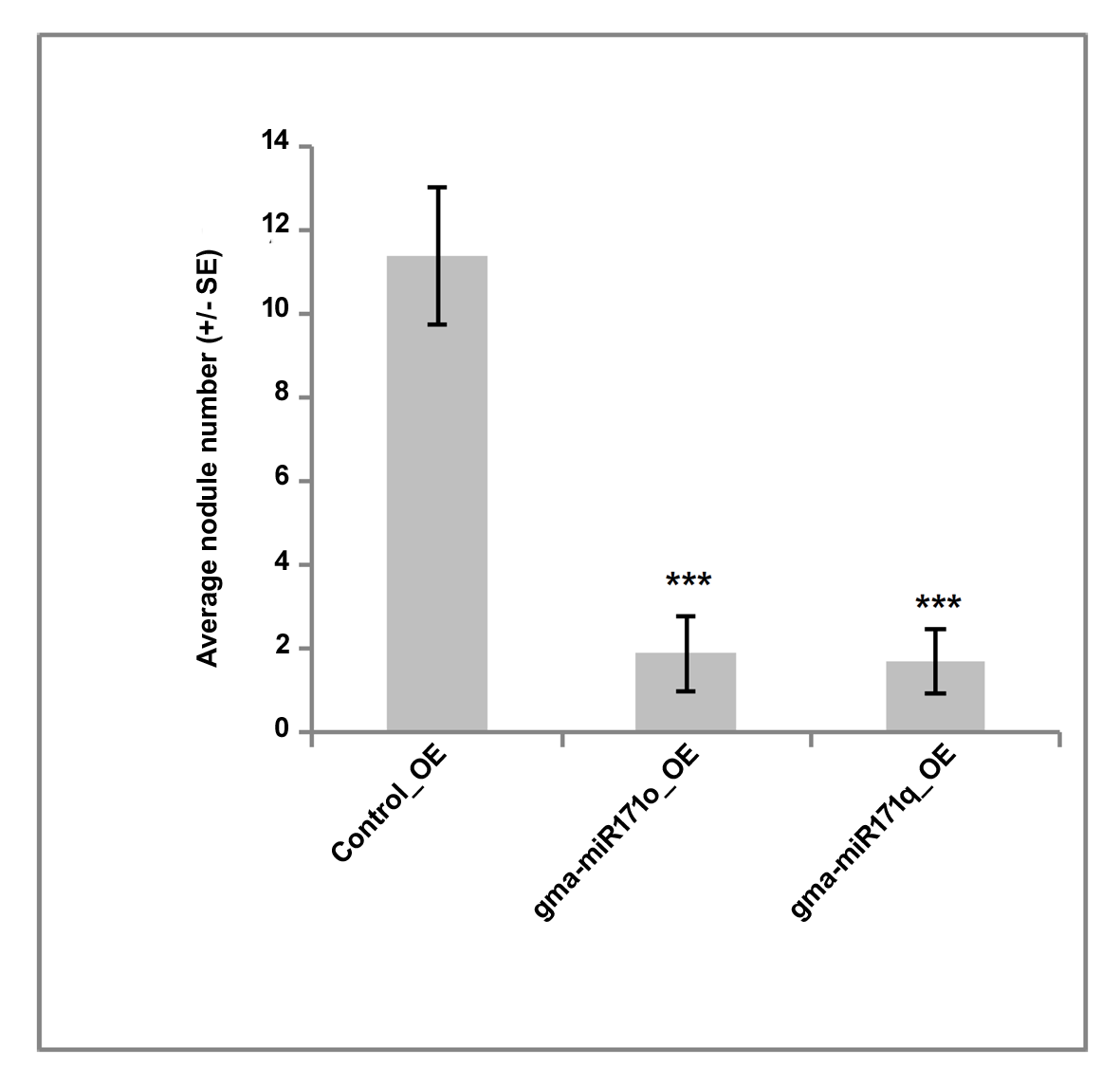

Supplement: Figure S2 — Average nodule number formed on transgenic hairy roots at 4 wpi by ectopic expression of gma-pri-miR171o and gma-pri-miR171q. Nodule number was counted from at least 20 transgenic hairy roots from two independent experiments. ∗∗∗indicate t-test significance of P < 0.0005. [file Image_2.TIF]

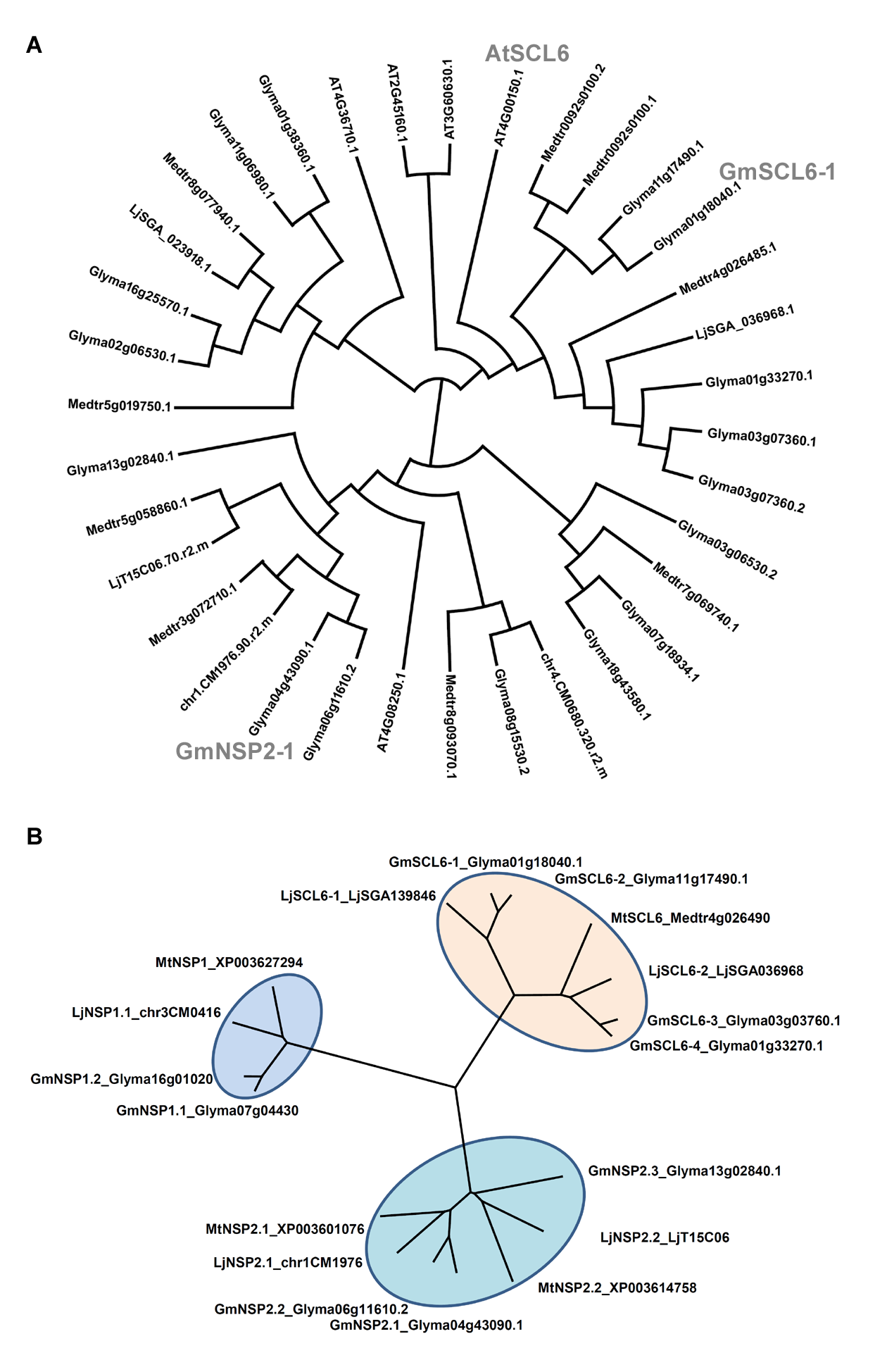

Supplement: Figure S3 — Soybean contains multiple copies of GmNSP2 and GmSCL6 proteins and they are phylogenetically distinct member of GRAS super family proteins. (A) Phylogenetic tree of GmNSP2 and GmSCL6 and their homologs in Arabidopsis, L. japonicus and M. truncatula. (B) Phylogenetic relationship of soybean homologs of GmNSP1, GmNSP2, and GmSCL6 along with proteins from L. japonicus and M. truncatula. [file Image_3.TIF]

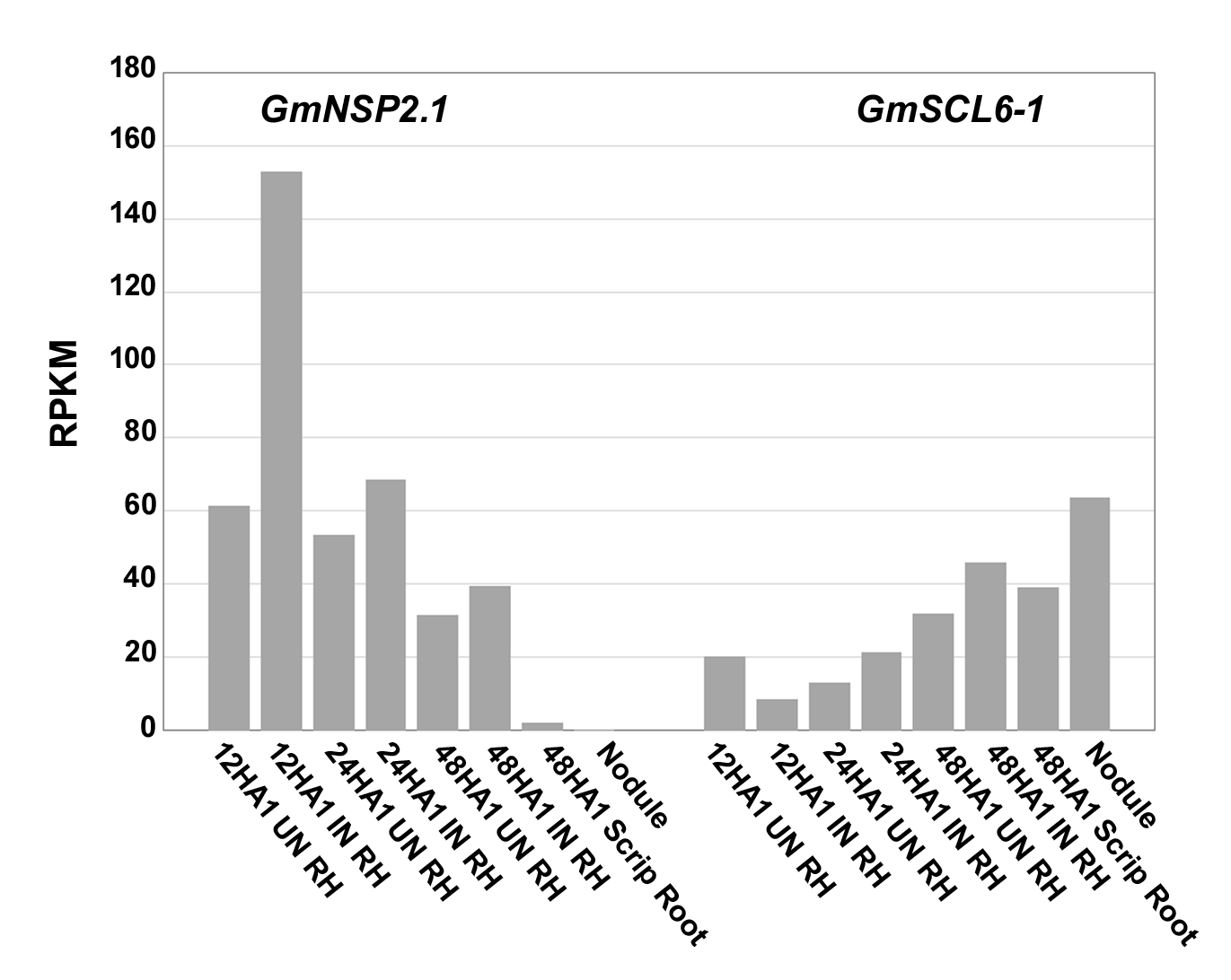

Supplement: Figure S4 — Expression patterns of GmNSP2.1 and GmSCL6-1 in early stage of soybean root hair infection and the later stage of nodules after B. japonicum inoculation. RPKM values from different time points as indicated below were collected from the SoyKB database (soykb.org). [file Image_4.TIF]
