# Supplementary material for: Characterization of the Spatial and Temporal Expression of Two Soybean miRNAs Identifies SCL6 as a Novel Regulator of Soybean Nodulation
Source: Front Plant Sci. 2019 Apr 16;10:475. doi: 10.3389/fpls.2019.00475 (PMC6477095; doi:10.3389/fpls.2019.00475)
Supplement: Table S1 — Primer used for this study. [file Data_Sheet_1.PDF]

Supplementary Table 1: Primer used for this study

**Stem loop RT-primers:**	**Sequences**	**Comments**	**References**
gma-miR171o and gma-miR171q:	GTCGTATCCAGTGCAGGGTCCGAGGTATTCGCACTGGATACGACTGTGAT	RT primer	
gma-miR171o and gma-miR171q	GGCGTTGAGCCGTGCCAAT	Forward	
Universal Reverse primer	AGTGCAGGGTCCGAGGTATT	Reverse	Zhe et al. 2013; 2015
5.8S_Forward	GCCTGCCTGGGTGTCACAC	Internal Standard	Zhe et al. 2013; 2015
5.8S_Reverse	GCGAGCACAGAATTAATACGAC	Internal Standard	Zhe et al. 2013; 2015
snoR1_Forward	GAAGATGAAGAGCTTTGTATATTC	Internal Standard	Zhe et al. 2013; 2015
snoR1_Reverse	ACTCAGAGAGTTGCTTTCTGTG	Internal Standard	Zhe et al. 2013; 2015
**miRNA overexpression cloning primers:**			
gma-miR171o-F	GTCACATATGTTTCATTAACGAA	Forward	
gma-miR171o-R	GATCTCAGTATTAAAGGGTAATA	Reverse	
gma-miR171q-F	ATGGATATATAGGAGTGTTACC	Forward	
gma-miR171q-R	ACCTTTATGGTGCTGCAAAGT	Reverse	
**precursors qRT-PCR primers:**			
gma-miR171c	CTTTAGAAAGCGCAGGAATGA	Forward	
gma-miR171f	TTGATGGGGTTCTCTACAACA	Forward	
gma-miR171o	ATGTTTCATTAACGAATGAATGG	Forward	
gma-miR171q	AGGATCAAACTTCTAACGAATAGC	Forward	
gma-miR171-rev	TTGGCACGGCTCAATCAAAT	common Reverse for c, f, o and q	
**qRT-PCR primers for target genes:**			
GmSCL6-1	GCACATGTTGTAAGCTCAATCTG	Glyma01g18040-F	
GmSCL6-1	ACTAACTGAAGAATTCCCATCCCA	Glyma01g18040-R	
GmSCL6-2	TCCACGGATAGCACGACAG	Glyma11g17490-F	
GmSCL6-2	CATTCCCAGCCCACATCTTG	Glyma11g17490-R	
GmSCL6-3	ACCACAGACAAAGCGGACTAT	Glyma01g33270-F	
GmSCL6-3	GCTGCTGCCAAGAGAAGAG	Glyma01g33270-R	
GmSCL6-4	GAATCGTTGGATGCGGTGAAT	Glyma03g03760-F	
GmSCL6-4	TTCTTGATAGCTGGCTGGATGA	Glyma03g03760-R	
GmNSP2.1	CACGACGACTTCCACCACC	Glyma04g43090-F	
GmNSP2.1	CCGGTTTCTTCGTCGGCGT	Glyma04g43090-R	
GmNSP2.2	TCCTCTGCTTGTTGTCGAGC	Glyma13g02840-F	
GmNSP2.2	GACACTCCATTTCATGTCAACCC	Glyma13g02840-R	
GmNSP2.3	CAATAGCACCACGCCCCCC	Glyma01g38360-F	
GmNSP2.3	TGGTGACGAGAACGGAGTG	Glyma01g38360-R	
GmNSP2.4	GCGAGAATACCACCGTGCT	Glyma11g06980-F	
GmNSP2.4	ATCCCTCACCGTCCACGAA	Glyma11g06980-R	
**miRNA Precursors promoter cloning primers:**			
Pro-gma-miR171o-F	AGGTCACAGGTCACAGGTCA	Forward	
Pro-gma-miR171o-R	TCATCAGCAAGTTCCTGCAC	Reverse	
Pro-gma-miR171q-F	AGAGCTAAGCTGCCCAATCA	Forward	
Pro-gma-miR171q-R	AGTTTGATCCTACACACTTCC	Reverse	
**miRNA targets promoter cloning primers:**			
pGmNSP2.1-F1	CCTCGCGGGGTCTTCAAATA	Forward	
pGmNSP2.1-R1	AAGTGGTGTTGTGTGGGGAG	Reverse	
pGmSCL6-1-F1	CGCCAAGTCGATGTGATGAC	Forward	
pGmSCL6-1-R1	AAATCAACCCCGACAAGTTG	Reverse	
**Mutated version of precursors cloning primers:**			
gma-miR171o-F1	GTCACATATGTTTCATTAACGAA	Forward 1	
gma-miR171o-mR1	TGTGATATTGTTCCGGCTCAA	mutant reverse 1	
gma-miR171o-mF2	TTGAGCCGGAACAATATCACA	mutant Forward 2	
gma-miR171o-R2	GATCTCAGTATTAAAGGGTAATA	mutant reverse 2	
gma-miR171q-F1	ATGGATATATAGGAGTGTTACC	Forward 1	
gma-miR171q-mR1	TGTGATATTGTTGCGGCTCAA	mutant reverse 1	
gma-miR171q-mF2	TTGAGCCGCAACAATATCACA	mutant Forward 2	
gma-miR171q-R2	ACCTTTATGGTGCTGCAAAGT	mutant reverse 2	
**Mutated version of precursor target cloning primers:**			
GmSCL6-1_F1	ATGAAGGCCGTGCCCCTACCCT	Forward 1	
GmSCL6-1_mR1	TTGAGCCGCATGAATATCCCA	mutant reverse 1	
GmSCL6-1_mF2	TGGGATATTCATGCGGCTCAA	mutant Forward 2	
GmSCL6-1_R2	TTAGCATCTCCAAGTTGAAACTG	mutant reverse 2	
GmNSP2.1_F1	ATGGAAATAGACATGGACATG	Forward 1	
GmNSP2.1_mR1	TTGAGCCGATATAATATCACT	mutant reverse 1	
GmNSP2.1_mF2	AGTGATATTATATCGGCTCAA	mutant Forward 2	
GmNSP2.1_R2	TTAAAATGAATTAATCTGAGTCC	mutant reverse 2	
**miRNA target RNAi primers:**			
GmSCL6-1-RNAi-F	TCTCCCTCTCTGGCACATGTT	Glyma01g18040	
GmSCL6-1-RNAi-R	AGAATTCCCATCCCATTCCA	Glyma01g18040	
GmNSP2.1-Rnai-F	TTTCTGCCTCCCTTTGGACTCA	Glyma04g43090	
GmNSP2.1-Rnai-R	AAGAGGTTGCTTTAGGCAAAGA	Glyma04g43090	
**Cloning primers for target proteins fusion:**			
GmNSP1.1-F	CAATGATCATGGAACCAAACCCAACA	Glyma07g04430	n-terminal
GmNSP1.1-R	CTGTTGAGCTCTGATCATTCCCA		c-terminal reverse without stop codon
GmNSP2.1-F	CCATGGAAATAGACATGGACATGGACT	Glyma04g43090	n-terminal
GmNSP2.1-R	CAAATGAATTAATCTGAGTCCAAA		c-terminal reverse without stop codon
GmSCL6-1-F	CAATGAAGGCCGTGCCCCTACCCTT	Glyma01g18040	n-terminal
GmSCL6-1-R	CGCATCTCCAAGTTGAAACTGAGA		c-terminal reverse without stop codon
**qRT-PCR primers for early nodulin genes:**			
GmENOD40a-F	TCTCTCTTGAGTGGCAGAAGCA	Glyma01g03470	Satomi et al. (year)
GmENOD40a-R	TGGAGTCCATTGCCTTTTCG		Satomi et al. (year)
GmENOD40b-F	GAGTGGCGGAAGCAGATACAC	Glyma02g04180	Satomi et al. (year)
GmENOD40b-R	CTACATAGCCATAGAGACCCCAATG		Satomi et al. (year)
GmNIN1a-F	TGGCGCACCATGCTAACAT	Glyma04g00210	Satomi et al. (year)
GmNIN1a-R	GGGTGTCATGGCAATCCTTT		Satomi et al. (year)
GmNIN2a-F	CAAGCCCTGTTTTGCTATGGA	Glyma02g48080	Satomi et al. (year)
GmNIN2a-R	TAGAGGAATAGCGAC GGCAGAA		Satomi et al. (year)
GmNIN2b-F	ACAGGGATGCTGGGTGGAA	Glyma14g00470	Satomi et al. (year)
GmNIN2b-R	CGCAGCCTCTAGTCTTTCTTTGA		Satomi et al. (year)
GmERN1a-F	TGCCTATAAACCAGATTTGAGCAA	Glyma19g29000	Satomi et al. (year)
GmERN1a-R	TCGAATATTTGAGCCATAGGGAAA		Satomi et al. (year)
GmERN1b-F	CCGCAAATACTCGAAGTTCGA	Glyma16g04410	Satomi et al. (year)
GmERN1b-R	CAGTTCCAAAGCCCCATGAA		Satomi et al. (year)
Gm ATP syn-F	GCGATTCTTAAGCCAGCCTTT	Glyma20g25920	Satomi et al. (year)
Gm ATP syn-R	ACACACCCTGGAAACTGGTGA		
Cons4-F	GATCAGCAATTATGCACAACG	BU578186	Libault et al. 2008
Cons4-R	CCGCCACCATTCAGATTATGT		Libault et al. 2009
Cons6-F	AGATAGGGAAATGGTGCAGGT	CD397253	Libault et al. 2010
Cons6-R	CTAATGGCAATTGCAGCTCTC		Libault et al. 2011
